# Influence of Thermal Treatment and Particle Size on the Physicochemical Properties and Filler Performance of Oyster Shell-Derived CaCO_3_ in Mortar

**DOI:** 10.3390/ma19081656

**Published:** 2026-04-21

**Authors:** Jessica de Dios-Suárez, Brayan Leonardo Pérez-Escobar, Germán Pérez-Hernández, Francisco Iván Lizama-Tzec, Laura Lorena Díaz-Flores, Salatiel Pérez-Montejo, Juan Pablo de Dios-Jiménez, Rafael Torres-Ricárdez

**Affiliations:** 1 División Académica de Ingeniería y Arquitectura, Universidad Juárez Autónoma de Tabasco, Av. Universidad S/N, Zona de la Cultura, Col. Magisterial, Villahermosa 86040, Mexico; 2 Departamento de Física Aplicada, Centro de Investigación y de Estudios Avanzados del Instituto Politécnico Nacional, Merida 97310, Mexico; 3 Facultad de Ingeniería, Universidad Autónoma del Carmen, Campus III, Av. Central S/N, Esq. Con Fracc. Mundo Maya, Ciudad del Carmen, Campeche 24115, Mexico; sperez@pampano.unacar.mx

**Keywords:** calcium carbonate, oyster shell waste, cementitious materials, compressive strength, thermal treatment

## Abstract

**Highlights:**

- Thermal treatment at 600 °C modified the CaCO_3_ texture, increasing the specific surface area without phase transformation.
- Calcination reduced the crystallite size and increased the surface area (5.8 → 25.6 m^2^/g).
- Compressive strength remained comparable to the reference mortar (13.6 MPa), with variations generally within ±10%.
- The highest strength (15.0 MPa) was achieved using finer calcined CaCO_3_ particles.
- Particle size and thermal treatment jointly control filler performance in mortar systems.
- Oyster shell-derived CaCO_3_ is a viable 10 wt.% cement replacement for non-structural applications.

**Abstract:**

The cement industry contributes approximately 7–8% of global CO_2_ emissions, motivating the development of sustainable supplementary materials. This study evaluates the partial replacement (10 wt.%) of Portland cement with calcium carbonate (CaCO_3_) derived from oyster shells, both untreated and thermally treated at 600 °C, in non-structural mortar blocks. Structural and physicochemical characterization was performed using XRD, SEM, EDS, BET, and TGA to assess phase composition, morphology, and surface properties. Thermal treatment modified the textural characteristics of CaCO_3_, reducing the crystallite size and increasing the specific surface area (from 5.8 to 25.6 m^2^/g), without phase transformation. Compressive strength results, relative to a reference mortar (13.6 MPa), showed comparable performance, with variations generally within ±10%, although slightly larger deviations were observed for specific particle sizes. Finer calcined particles yielded the highest strength (15.0 MPa), reinforcing the combined influence of particle size and thermal treatment. These results suggest that CaCO_3_ acts primarily through a filler effect, improving particle packing and matrix interaction. Both untreated and heat-treated CaCO_3_ satisfied strength requirements for non-structural applications, supporting the valorization of oyster shell waste as a sustainable material in cement-based systems.

## 1. Introduction

The increasing demand for environmentally responsible construction materials has intensified the search for alternative mineral resources capable of reducing the carbon footprint of cement-based systems while maintaining mechanical performance and serviceability. Among these alternatives, oyster shell waste—an abundant calcium carbonate-rich byproduct of the agri-food industry—has gained attention as a potential supplementary material within circular economy strategies. Due to its high CaCO_3_ content and availability, this waste has been increasingly explored as a mineral addition in cement-based systems, making it a promising candidate for incorporation into mortar and concrete formulations aimed at reducing clinker consumption [1].

Concrete remains the most widely used construction material worldwide [2]; however, Portland cement production is responsible for significant CO_2_ emissions [3,4] and substantial energy consumption [5]. Strategies aimed at partially replacing clinker with mineral additions or waste-derived fillers have therefore become essential for mitigating environmental impact. Among these, calcium carbonate-based materials have been widely studied due to their ability to improve particle packing and contribute to early hydration processes, making them a viable alternative for sustainable cementitious systems.

Oyster shells typically constitute 60–90% of the total mass of bivalves and are predominantly composed of CaCO_3_ in calcite and aragonite polymorphic forms [6,7,8]. In cementitious systems, calcium carbonate is generally recognized for its physical filler effect, improving particle packing and acting as nucleation sites for hydration products. Additionally, under certain conditions, CaCO_3_ may interact with aluminate phases to form carboaluminate compounds, potentially modifying microstructural development [3,9]. These effects are strongly dependent on particle size distribution, surface characteristics, and mineralogical composition, which govern its interaction with the cementitious matrix.

Previous investigations have reported improvements in compressive and flexural strength when finely ground oyster shell waste is incorporated into cementitious mixtures, primarily attributed to filler densification and enhanced hydration kinetics [1,10,11]. For instance, Chen et al. [12] evaluated crushed oyster shell mortars incorporating supplementary cementitious materials and demonstrated their potential to improve mechanical performance and sustainability, with compressive strength values in the range of approximately 12–35 MPa, depending on the mixture composition. It should be noted that these values were obtained using a cement-to-sand ratio of 1:3, which typically results in higher strength due to the greater cement content. Liao et al. [13] investigated the influence of calcination temperature on oyster shell powder and observed that thermal treatment significantly affects reactivity, with compressive strength values varying between 10 and 35 MPa under different processing conditions. In contrast, the present study employs a cement-to-sand ratio of 1:4, which may contribute to comparatively lower strength values and should be considered when interpreting the results.

Similar performance ranges have also been reported for other calcium-rich residues, such as limestone powder and eggshell-derived CaCO_3_, confirming their viability as supplementary materials in non-structural applications. However, biogenic CaCO_3_ from oyster shells exhibits distinctive morphological features and polymorphic distributions that may influence its packing behavior and surface reactivity [8,14].

Despite these advances, previous studies have primarily focused on finely ground particles or on calcination effects evaluated independently. In contrast, the combined influence of particle size distribution—including both fine and coarse fractions—and controlled sub-decarbonation thermal treatment on the physicochemical characteristics and mechanical performance of biogenic CaCO_3_ remains insufficiently explored. This gap is particularly relevant, as particle size governs the transition between filler-like and aggregate-like behavior, directly influencing the performance of non-structural mortar systems.

Based on the above considerations, this study evaluates the influence of particle size distribution and controlled thermal treatment (600 °C) on the physicochemical characteristics of oyster shell-derived CaCO_3_ and its performance as a supplementary material incorporated at 10 wt.% relative to cement, aiming to evaluate its size-dependent filler behavior in mortar blocks. It is hypothesized that thermal treatment below the decarbonation threshold modifies surface characteristics and apparent crystallite size of biogenic CaCO_3_, thereby enhancing its filler efficiency, particularly for finer particle fractions. The investigation focuses on structural characterization and compressive strength performance at 28 days to assess the practical feasibility of valorizing oyster shell waste in non-structural cement-based applications.

## 2. Materials and Methods

**Figure 1:**
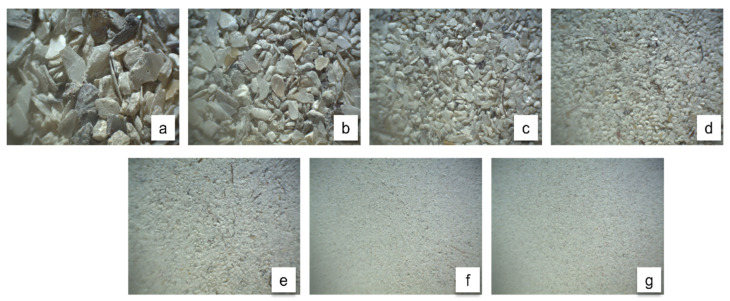
Microscopic images of CaCO_3_ particles at 4.5× magnification. Particle sizes: 0.84 mm (**a**), 0.42 mm (**b**), 0.25 mm (**c**), 0.17 mm (**d**), 0.15 mm (**e**), 0.07 mm (**f**), and 0.04 mm (**g**).

Oyster shell waste from Crassostrea virginica collected in the Mecoacán Lagoon (Paraíso, Tabasco, Mexico) was used as the CaCO_3_ source. The shells were manually washed with tap water, mechanically brushed to remove adhering organic matter, and then air-dried for 72 h under ambient conditions until no visible moisture was observed and mass stabilization was achieved. The dried shells were crushed using a mechanical mill, producing particles between 0.04 and 0.84 mm (Figure 1). Particle size classification was carried out using sieves compliant with ASTM E11 [15], with mesh sizes of 20, 40, 60, 80, 100, 200, and 325, corresponding to aperture sizes of 0.84 mm, 0.425 mm, 0.25 mm, 0.18 mm, 0.15 mm, 0.075 mm, and 0.045 mm, respectively

Notably, the particle size range used in this study (0.04–0.84 mm) extends beyond the typical size of cement particles (<45 µm). The selection of this broad granulometric range was intentional and aimed at evaluating the size-dependent behavior of biogenic CaCO_3_ when incorporated into mortar systems. While finer fractions (<75 µm) are expected to behave as conventional mineral fillers, contributing to particle packing and surface interactions, coarser fractions may act as fine aggregate-like inclusions, influencing matrix heterogeneity and load transfer mechanisms. Therefore, the present study does not address the chemical replacement of cement but rather investigates the combined effects of particle-size distribution and thermal treatment on the performance of CaCO_3_ as a supplementary material in non-structural mortars. Similar approaches have been reported in studies involving limestone powders and calcium-rich waste materials, where the effect of particle size distribution has been shown to influence packing behavior, hydration, and mechanical performance. In particular, finer particles typically act as mineral fillers enhancing surface interactions, while coarser fractions may behave as fine aggregate-like inclusions, contributing differently to matrix structure and strength development [16,17,18].

Thermal treatment was conducted at 600 °C for 2 h in air to remove residual organic matter and promote the structural reorganization of biogenic CaCO_3_ while preventing decarbonation to CaO, which typically occurs at higher temperatures (>700 °C) [19,20]. The treatment was conducted in air to facilitate the removal of organic residues and to simulate conventional processing conditions for mineral fillers. This temperature was selected based on reported stability ranges of calcite, ensuring preservation of the primary crystalline phase while potentially modifying surface characteristics relevant to filler performance. Phase identification was carried out by X-ray diffraction (XRD) using a Bruker D8 ADVANCE diffractometer (Bruker AXS GmbH, Billerica, MA, USA) with Cu Kα radiation (λ = 1.5406 Å), scanning from 20° to 60° (2θ) at a 0.02° step size. Elemental composition and particle morphology were examined via energy dispersive X ray spectroscopy (EDS) and scanning electron microscopy (SEM) on a FE-SEM; JEOL JSM-7600F (JEOL, Tokyo, Japan) operating at 20 kV. Thermal stability was evaluated through thermogravimetric analysis (TGA) using a Discovery TGA 5500 (TA Instruments, New Castle, DE, USA) under dry air flow (50 mL/min), heating from 25 °C to 550 °C at 10 °C/min. The specific surface area and pore size distribution were measured by N_2_ adsorption using the BET method on a BELSORP max system (MicrotracBEL Corp., Osaka, Japan) after degassing samples at 220 °C for 3 h.

Mortar mixtures were prepared by incorporating CaCO_3_ at a fixed proportion of 10 wt.% relative to the cement mass to evaluate its behavior as a supplementary material across varying particle sizes and mechanical response under controlled conditions. This approach allows assessment of the combined influence of fine and coarse fractions on the physical performance of the mortar rather than representing a direct replacement of cement with particles of equivalent size. The selected replacement level of 10 wt.% was based on previous studies reporting that partial substitution of cement with calcium carbonate-based materials within the range of 5–15 wt.% provides a balance between maintaining mechanical performance and enhancing filler effects [17,21,22]. Liao et al. [13] demonstrated that a ~10 wt.% substitution with calcined oyster shell powder (950 °C) optimizes CaO reactivity and compressive strength while maintaining adequate workability. Several authors have reported that replacement levels around 10 wt.% enhance particle packing and hydration kinetics while maintaining comparable compressive strength [17,22]. Additionally, this proportion has been widely adopted in studies involving oyster shell-derived CaCO_3_ and other calcium-rich residues, facilitating comparison with existing literature. Therefore, the selected dosage enables evaluation of the combined influence of particle size and thermal treatment under representative and technically relevant conditions.

This replacement ratio was selected to enable comparative analysis of filler performance while maintaining workability and structural integrity consistent with non-structural applications. Mortar mixtures were prepared using Portland cement from the CEMEX brand, compliant with NMX-C-414-ONNCCE [23], functionally equivalent to an ASTM C150 [24], Type I cement, river sand, and tap water. A cement-to-sand ratio of 1:4 and a water-to-cement ratio (*w*/*c*) of 0.68 were used, following the procedure adapted from Mora Ortiz et al. (2020) [25]. The cement-to-sand ratio of 1:4 was selected based on previous studies and is representative of non-structural mortar applications, where reduced cement content is commonly used to balance mechanical performance and material sustainability. Cylindrical specimens (2 in. in diameter and 4 in. in height) were prepared following a procedure based on ASTM C192 [26], adapted for mortars, and cured for 28 days at 25 ± 2 °C and 95% relative humidity. Untreated and calcined CaCO_3_ particles were incorporated at sizes of 0.84, 0.42, 0.25, 0.17, 0.14, 0.07, and 0.04 mm. Compressive strength was measured on a Siemens universal testing machine under displacement control at 1.5 mm/min. The procedure was based on the general principles of ASTM C39 [27], adapted for mortars and displacement control. All tests were conducted in triplicate.

## 3. Results and Discussion

**Figure 2:**
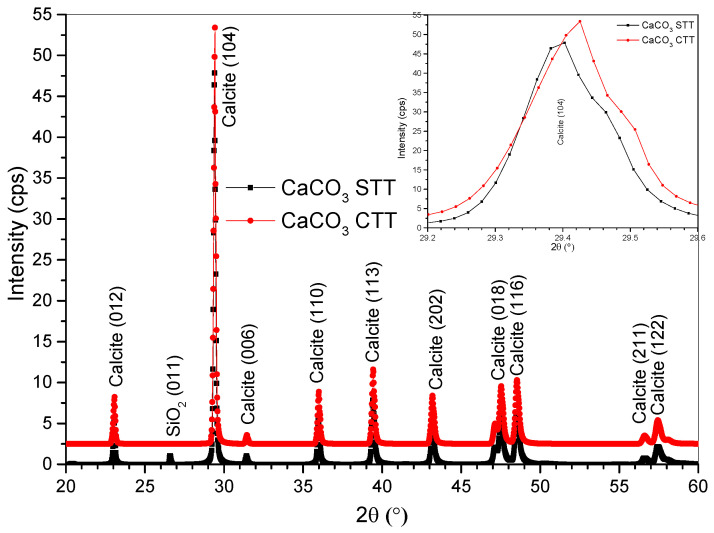
X-ray diffraction patterns of CaCO_3_, untreated and thermally treated at 600 °C.

The X-ray diffraction patterns of untreated and heat-treated CaCO_3_ (Figure 2) confirm that calcite is the predominant crystalline phase, with characteristic reflections at (012) (2θ ≈ 23.1°), (104) (2θ ≈ 29.4°), (110) (2θ ≈ 36.0°), (113) (2θ ≈ 39.4°), (202) (2θ ≈ 43.2°), (018) (2θ ≈ 47.6°), (116) (2θ ≈ 48.6°), and (122) (2θ ≈ 57.5°). A weak SiO_2_ reflection at (011), detected in the untreated sample, disappears after calcination, indicating the removal or transformation of minor silica-containing impurities [28]. Upon thermal treatment at 600 °C, relative increases in the intensities of the (113), (202), (116), and (122) reflections are observed. These changes, together with texture coefficients exceeding unity (TC > 1) [29] for specific planes (Table 1), indicate modifications in crystallographic orientation distribution rather than phase transformation. No additional diffraction peaks associated with CaO or secondary crystalline phases were detected within the analyzed range, supporting the preservation of calcite under the applied thermal conditions. Similar behavior has been reported for biogenic CaCO_3_ subjected to sub-decarbonation thermal treatments, in which defect reduction and lattice rearrangement promote the selective growth of specific crystallographic planes without phase transformation [19,30].

The crystallite size, estimated using the Debye–Scherrer equation (K = 0.9) [31] from the (104) reflection, decreased from approximately 74 nm in the untreated sample to about 57 nm after calcination. This variation indicates a reduction in the coherent diffraction domain size following thermal treatment at 600 °C. While the Scherrer approach does not distinguish between crystallite refinement and microstrain effects, the observed decrease is consistent with thermally induced modification of lattice coherence occurring below the decarbonation temperature of calcite. No additional diffraction peaks corresponding to CaO or other crystalline phases were detected within the analyzed angular range, suggesting phase stability under the applied conditions. These structural modifications, together with the increased surface area observed in BET analysis, may influence the filler behavior of the material when incorporated into cementitious matrices.

**Figure 3:**
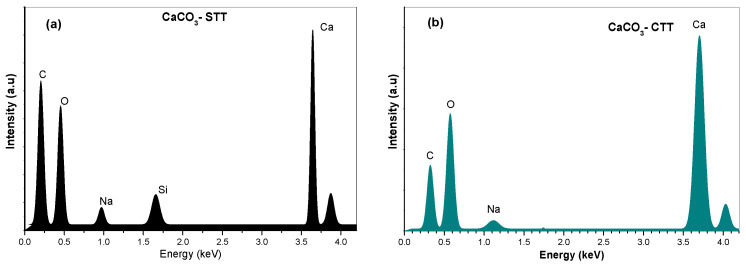
EDS analysis curves of CaCO_3_ that was untreated (**a**) and heat-treated at 600 °C (**b**).

As shown in Figure 3a,b, the EDS spectra confirm that both untreated and calcined CaCO_3_ samples are primarily composed of calcium, oxygen, and carbon. The spectra exhibit characteristic peaks corresponding to Ca (≈3.7 keV), O (≈0.5 keV), and C (≈0.3 keV), revealing that the material is predominantly composed of calcium, oxygen, and carbon, consistent with CaCO_3_ as identified by XRD analysis. The semi-quantitative analysis indicates calcium (12.8–12.3%), oxygen (55.2–47.9%), and carbon (29.2–38.0%), with silicon detected in minor amounts (<1.2%), suggesting the presence of trace mineral impurities. A comparison between the EDS spectra shows slight variations in peak intensities, particularly for oxygen and carbon. However, these variations should be interpreted with caution due to the limited quantitative accuracy of EDS for light elements and its sensitivity to measurement conditions. These changes may be qualitatively associated with the removal of surface-bound moisture and organic residues after thermal treatment, as supported by TGA results. Overall, the elemental distribution and the presence of dominant Ca, O, and C peaks indicate that the material composition remains consistent after thermal treatment within the detection limits of the technique. No additional elemental signals were detected in the EDS spectra, demonstrating that the elemental composition remained consistent after thermal treatment. These observations are aligned with XRD results, which confirmed the absence of phase transformation under the applied thermal conditions.

**Figure 4:**
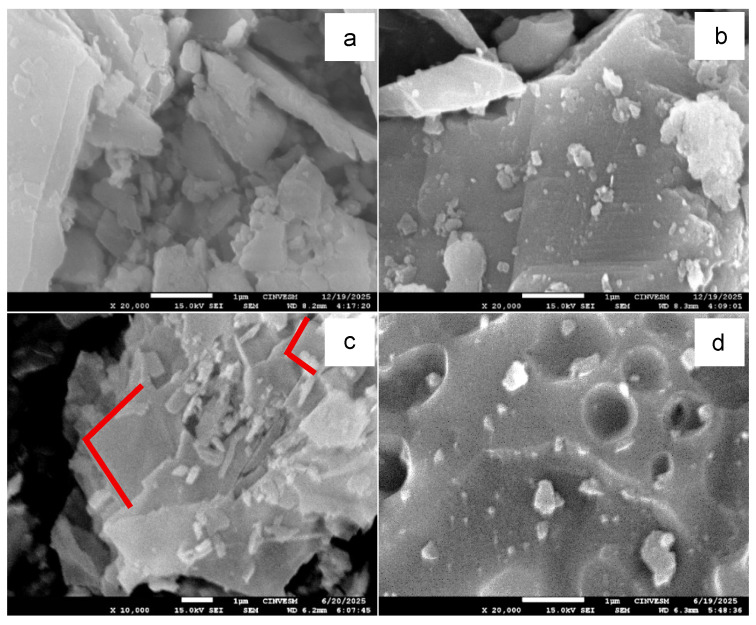
SEM images show the morphology of CaCO_3_ particles that were untreated and heat-treated at 600 °C. (**a**) STT; (**b**) CTT; (**c**) STT; (**d**) CTT.

SEM micrographs (Figure 4) reveal flat and elongated CaCO_3_ particles in both untreated and calcined samples (Figure 4a,b), together with well-defined rhombohedral calcite crystals with sizes in the range of 2–4 µm (Figure 4c), consistent with the XRD identification of calcite as the main crystalline phase. After calcination, the material exhibits increased particle agglomeration and apparent fragmentation of larger particles. Additionally, the presence of rounded void-like features in the heat-treated sample (Figure 4d) suggests localized structural modification, possibly associated with the removal of organic constituents naturally present in biogenic CaCO_3_. These morphological changes are qualitatively consistent with the mass loss observed in TGA and the increase in specific surface area measured by BET, indicating that thermal treatment influences surface characteristics relevant to filler behavior.

**Figure 5:**
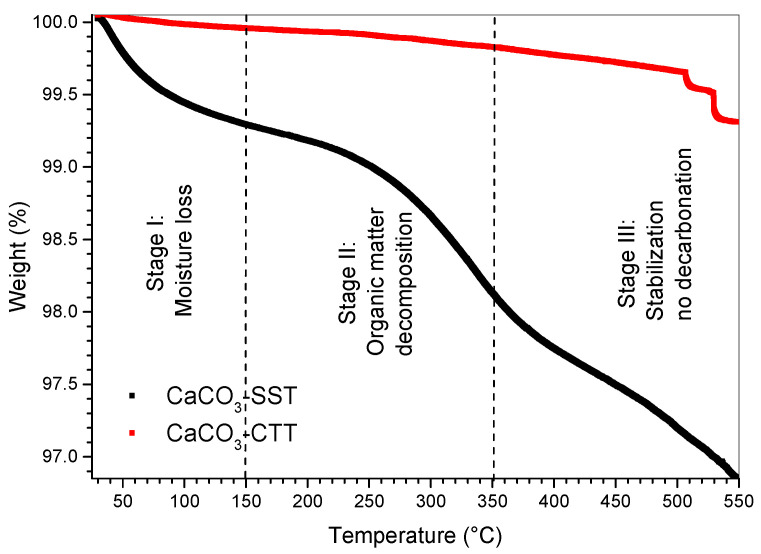
Thermogravimetric analysis (TGA) curves of CaCO_3_ that was untreated and heat-treated at 600 °C.

TGA results (Figure 5) indicate that CaCO_3_ exhibits high thermal stability up to 550 °C, with total mass losses of 3.15% for the untreated sample and 0.7% for the calcined material. The thermogravimetric curves reveal three distinct stages associated with different thermal processes. In the first stage (25–150 °C), a minor mass loss is observed, attributed to the removal of physically adsorbed moisture. In the second stage (150–350 °C approximately), a gradual mass loss occurs, associated with the decomposition of residual organic matter inherent to the biogenic structure. Beyond this range (350–550 °C), the curves exhibit negligible mass loss, indicating a thermally stable region, confirming the absence of significant decomposition processes within the studied temperature range. These stages are consistent with previous studies on biogenic calcium carbonate and similar calcium-rich materials, where moisture removal and decomposition of organic matter have been reported within comparable temperature intervals [19,32,33,34].

No pronounced mass-loss event corresponding to CaCO_3_ decarbonation (typically occurring above 700 °C) was observed, confirming the thermal stability of calcite within the analyzed temperature range. The lower mass loss recorded for the calcined sample suggests that partial dehydration and organic matter removal occurred during the prior thermal treatment at 600 °C. This behavior explains the reduced volatile content of the treated material. These observations are consistent with the increase in specific surface area observed in BET analysis, indicating that thermal treatment modifies surface characteristics relevant to filler performance.

**Figure 6:**
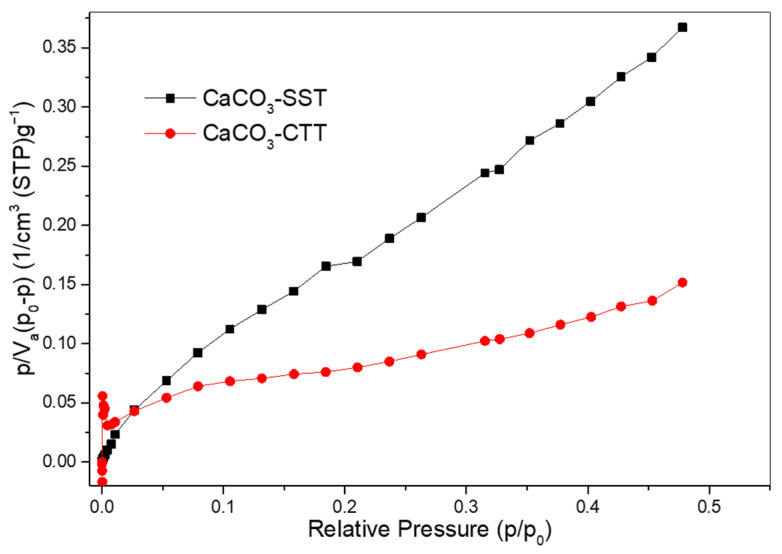
BET linear plot of [p/V_a_(p_0_ − p)] versus relative pressure (p/p_0_) for CaCO_3_ that was untreated and heat-treated at 600 °C.

BET analysis (Figure 6) showed that thermal treatment at 600 °C significantly modified the textural properties of CaCO_3_, reducing the average pore diameter from 17.5 nm to 5.0 nm and increasing the specific surface area from 5.8 to 25.6 m^2^/g. This substantial increase in specific surface area suggests the development of finer porosity and enhanced surface exposure following calcination. Such textural modification is consistent with the removal of organic constituents identified by TGA and with the structural changes inferred from XRD and SEM observations. Similar increases in the surface area and pore refinement have been reported for biogenic CaCO_3_ subjected to sub-decarbonation thermal treatments [35]. The higher specific surface area may contribute not only to improved filler efficiency by enhancing particle–matrix contact and packing density, but also to increased surface reactivity, promoting nucleation processes and potential interactions with cement hydration products [13,36,37].

**Figure 7:**
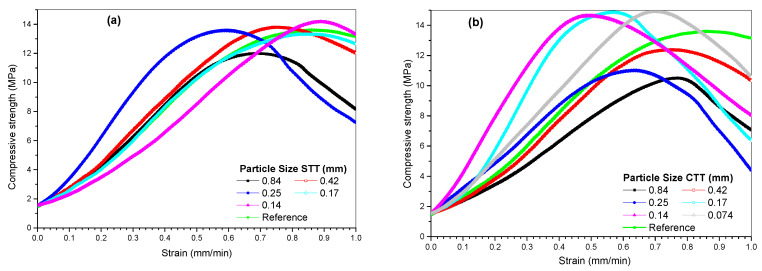
Compressive strength of mortar blocks prepared with CaCO_3_: heat-treated (**a**) and un-treated (**b**) samples.

Compressive strength results (Figure 7) show a consistent increase in strength with decreasing CaCO_3_ particle size for both untreated and calcined materials. For comparison purposes, a control mortar without CaCO_3_ replacement was included, showing a compressive strength of approximately 13.6 MPa. This reference value serves as a baseline for evaluating the effect of CaCO_3_ incorporation, allowing a direct assessment of the influence of particle size and thermal treatment on mechanical performance. The incorporation of oyster shell-derived CaCO_3_ at 10 wt.% results in comparable or slightly reduced strength values depending on particle size and treatment condition. A quantitative comparison shows that the compressive strength of the modified mortars varies within a narrow range relative to the control (13.6 MPa), with differences generally within ±10%, although slightly larger deviations were observed for specific particle sizes (Table 2). For instance, the finest calcined fraction (0.074 mm) reached the highest strength, representing an increase of approximately 10% compared to the control, while coarser fractions exhibited slightly lower values. This quantitative comparison indicates that the observed variations are limited and does not indicate a systematic reduction in strength due to thermal treatment. In contrast, untreated samples showed more moderate variations, revealing that particle size plays a dominant role, while thermal treatment enhances performance primarily at finer scales. The maximum compressive strength recorded for untreated samples (STT) was approximately 14.1 MPa, while calcined samples (CTT) reached a maximum value of 15.0 MPa, as summarized in Table 2. This comparison highlights the slight improvement associated with thermal treatment, particularly at finer particle sizes. Overall, the variation in compressive strength remains limited, indicating that particle size has a more pronounced effect than thermal treatment alone. These results further indicate that the incorporation of oyster shell-derived CaCO_3_ does not significantly compromise mechanical performance.

Although statistical significance was not formally assessed, the observed trend suggests that finer particles contribute more effectively to the mechanical performance of the mortar. This behavior is consistent with the expected filler effect, whereby smaller particles may enhance packing density and reduce microvoids within the matrix. In particular, the optimized calcined fraction (0.074 mm) exhibited the best mechanical performance, indicating that particle size optimization can partially compensate for cement reduction. The absence of the untreated sample at 0.074 mm was a consequence of limitations in mechanical grinding, as untreated shells exhibited higher resistance to fragmentation compared to calcined material, which became more brittle after thermal treatment.

From a physico-chemical perspective, the incorporation of oyster shell-derived CaCO_3_ influences the mortar system primarily through physical and interfacial mechanisms. Fine CaCO_3_ particles act as microfillers that occupy voids between cement grains and sand particles, improving packing density and reducing porosity. Additionally, the increased specific surface area of calcined particles may provide additional sites that can promote interactions with hydration products and contribute to a more homogeneous microstructure. These effects are likely associated with improved stress transfer within the matrix and the observed mechanical performance.

The improved performance observed for calcined samples can also be qualitatively associated with their higher specific surface area, as determined by BET analysis, which may promote more effective particle–matrix interaction. In contrast, larger particles may act as inclusions that locally disrupt the continuity of the matrix, potentially limiting the efficiency of stress transfer. All evaluated mixtures meet the minimum compressive strength requirements for non-structural cement-based masonry units according to NMX-C-441-ONNCCE-2013 [38], confirming the technical viability of oyster shell-derived CaCO_3_ under the studied conditions.

## 4. Conclusions

Oyster shell-derived CaCO_3_ demonstrated physicochemical characteristics compatible with its use as a partial substitution of cement with size-dependent filler behavior in mortar systems. Thermal treatment at 600 °C modified the structural and textural properties of the material, as evidenced by changes in diffraction response, a reduction in coherent crystallite size, and a significant increase in the specific surface area. These modifications are indicative of alterations in surface characteristics without phase transformation.

The incorporation of CaCO_3_ particles resulted in compressive strength values comparable to those of the reference mortar (13.6 MPa), with variations generally within ±10%, although slightly larger deviations were observed for specific particle sizes, indicating that partial cement replacement does not significantly compromise mechanical performance. In particular, finer calcined particles exhibited the best performance, highlighting the importance of particle size optimization.

From a physico-chemical perspective, the observed behavior is associated with the filler effect of CaCO_3_ particles, which improves packing density and promotes more efficient stress transfer within the cementitious matrix. Although no direct porosity measurements were conducted, the observed strength enhancement is consistent with an enhanced filler effect associated with finer particle size and increased specific surface area.

Both untreated and calcined CaCO_3_ satisfied the compressive strength requirements for non-structural masonry applications, supporting the technical feasibility of valorizing oyster shell waste as a supplementary mineral resource in cement-based materials. These findings confirm that oyster shell-derived CaCO_3_ can be effectively incorporated without detrimental effects on mechanical performance when appropriate particle size and processing conditions are selected.

Regarding the limitations of the present experimental program, the results indicate that controlled thermal treatment and particle size selection are relevant parameters influencing filler performance in sustainable mortar formulations, particularly when evaluated in relation to a reference system and considering the combined effects of physical and interfacial mechanisms.

Future research should focus on evaluating the long-term performance and durability of mortars incorporating oyster shell-derived CaCO_3_, including permeability, shrinkage, and resistance to aggressive environments. Further studies should also address the influence of different replacement levels and particle size distributions. Additionally, advanced microstructural characterization is required to better understand the physico-chemical interactions governing the performance of biogenic CaCO_3_ in cementitious systems.

## Figures and Tables

**Table 1:** Texture coefficients of CaCO_3_ untreated and thermally treated at 600 °C.

Plane	Texture Coefficient CaCO_3_		Plane	Texture Coefficient CaCO_3_	
	STT	CTT		STT	CTT
(012)	0.90	0.89	(202)	0.98	1.30
(104)	1.47	1.27	(018)	1.48	1.27
(006)	1.64	1.30	(116)	0.95	1.18
(110)	0.45	0.59	(211)	0.63	0.88
(113)	0.93	1.04	(122)	0.76	1.26

Note. STT refers to the untreated sample, and CTT refers to the thermally treated sample.

**Table 2:** The maximum compressive strength obtained of mortar blocks prepared with CaCO_3_: heat-treated (CTT) and untreated (STT) samples.

Particle Size	Compressive Strength (MPa)	
	STT	CTT
Reference	13.6	13.6
0.84	12.0	10.5
0.42	13.8	12.3
0.25	13.6	10.9
0.17	13.3	15.0
0.14	14.1	14.7
0.074		15.0

## Data Availability

The original contributions presented in this study are included in the article. Further inquiries can be directed to the corresponding author.
